# Transportable hyperspectral imaging setup based on fast, high-density spectral scanning for *in situ* quantitative biochemical mapping of fresh tissue biopsies

**DOI:** 10.1117/1.JBO.29.9.093508

**Published:** 2024-09-10

**Authors:** Luca Giannoni, Marta Marradi, Kevin Scibilia, Ivan Ezhov, Camilla Bonaudo, Angelos Artemiou, Anam Toaha, Frédéric Lange, Charly Caredda, Bruno Montcel, Alessandro Della Puppa, Ilias Tachtsidis, Daniel Rückert, Francesco Saverio Pavone

**Affiliations:** a University of Florence, Department of Physics and Astronomy, Florence, Italy; b European Laboratory for Non-Linear Spectroscopy, Sesto Fiorentino, Italy; c Technical University of Munich, TranslaTUM - Center for Translational Cancer Research, Munich, Germany; d Azienda Ospedaliero-Universitaria Careggi, University of Florence, Neurosurgery, Department of Neuroscience, Psychology, Pharmacology and Child Health, Florence, Italy; e University College London, Department of Medical Physics and Biomedical Engineering, London, United Kingdom; f Université de Lyon, INSA-Lyon, Université Claude Bernard Lyon 1, UJM-Saint Etienne, CNRS, Inserm, CREATIS UMR 5220, Lyon, France; g Imperial College London, Department of Computing, London, United Kingdom; h National Research Council, National Institute of Optics, Sesto Fiorentino, Italy

**Keywords:** hyperspectral imaging, biomedical optics, biophotonics, digital histopathology, neurosurgery

## Abstract

**Significance:**

Histopathological examination of surgical biopsies, such as in glioma and glioblastoma resection, is hindered in current clinical practice by the long time required for the laboratory analysis and pathological screening, typically taking several days or even weeks to be completed.

**Aim:**

We propose here a transportable, high-density, spectral scanning-based hyperspectral imaging (HSI) setup, named HyperProbe1, that can provide *in situ*, fast biochemical analysis, and mapping of fresh surgical tissue samples, right after excision, and without the need for fixing, staining nor compromising the integrity of the tissue properties.

**Approach:**

HyperProbe1 is based on spectral scanning via supercontinuum laser illumination filtered with acousto-optic tunable filters. Such methodology allows the user to select any number and type of wavelength bands in the visible and near-infrared range between 510 and 900 nm (up to a maximum of 79) and to reconstruct 3D hypercubes composed of high-resolution (4 to 5  μm), widefield images ($0.9\times 0.9\;{\mathrm{mm}}^{2}$) of the surgical samples, where each pixel is associated with a complete spectrum.

**Results:**

The HyperProbe1 setup is here presented and characterized. The system is applied to 11 fresh surgical biopsies of glioma from routine patients, including different grades of tumor classification. Quantitative analysis of the composition of the tissue is performed via fast spectral unmixing to reconstruct the mapping of major biomarkers, such as oxy-(${\mathrm{HbO}}_{2}$) and deoxyhemoglobin (HHb), as well as cytochrome-c-oxidase (CCO). We also provided a preliminary attempt to infer tumor classification based on differences in composition in the samples, suggesting the possibility of using lipid content and differential CCO concentrations to distinguish between lower and higher-grade gliomas.

**Conclusions:**

A proof of concept of the performances of HyperProbe1 for quantitative, biochemical mapping of surgical biopsies is demonstrated, paving the way for improving current post-surgical, histopathological practice via non-destructive, *in situ* streamlined screening of fresh tissue samples in a matter of minutes after excision.

## 1 Introduction

Histopathological screening of excised tissue is the current “gold standard” in post-surgical oncological practice,^1^ for clinical and molecular evaluation of critical parameters such as type, grading, and classification of tumors, e.g., in glioma and glioblastoma (GBM) resection. ^2^^–^^4^ Normal routine involves the dispatch of fresh surgical biopsies after resection to the histopathology laboratory: therefore, the samples are typically fixed for preservation, sectioned, stained—various staining techniques are used, with hematoxylin and eosin (H&E) staining being the most prominent— and then imaged with a microscope to determine their structural and molecular composition.^3^ However, modern histopathological analysis presents several limitations, the most severe one being the lengthy preparation of the samples that lead to long duration of the procedures to obtain the final results, which can vary from several days to even weeks after the surgery. Extemporaneous and intraoperative analyses can be much faster (minutes to hours), but the number of biopsy samples is limited due to operational logistics and costs of the procedures, and the breadth of information they can provide is very limited for diagnostic purposes.^5^ Furthermore, diagnosis and classification can be affected by variability in the subjective interpretation of the results by histopathologists, with the screening essentially lacking a more quantitative and objective way to systematically process the imaging outcomes.^6^ Overall, postoperative prognosis and planning would enormously benefit from a different approach to histopathology that could provide much faster and more reliable information on the tissue biopsies, ideally by having a screening *in situ* right after the surgery that could lead to quantitative results in a matter of minutes to hours.

Hyperspectral imaging (HSI) is an optical imaging modality that is becoming increasingly more notable in recent years in the biomedical and bioimaging fields^7^ and whose main features can be particularly suited and advantageous to tackle the abovementioned challenge. HSI acquires and reconstructs images of a target at multiple, narrow, contiguous, or adjacent wavelength bands in the electromagnetic spectrum, typically spanning from the visible to the near-infrared (NIR) range.^8^ This allows the user to obtain 3D spatio-spectral datasets, named “hypercubes,” where each spatial pixel of the images is associated with a corresponding spectrum of reflected, transmitted, and/or fluorescent light. The information carried by the hypercubes is related to the optical properties of absorption and scattering of the investigated tissue, from which is then possible to infer, map, and quantify its biochemical and structural composition, without the need for time-consuming staining procedures or the use of any exogenous contrast agent. Intrinsic biomarkers for physiology and pathophysiology of the tissue can indeed be identified for diagnostic purposes, such as hemoglobin for hemodynamics, oxygenation and vascularization, or cytochrome-c-oxidase (CCO) for cellular metabolism,^8^^–^^10^ and related to tumor key parameters of classification.^11^ In addition to its capabilities for non-destructive biochemical analysis of freshly excised tissue, HSI has the additional advantage of fast image acquisition and data processing, mainly thanks to recent advancements in deep learning and artificial intelligence (AI) algorithms,^12^ achieving near real-time computing and almost immediate visualization of the results.^13^ Finally, HSI technology is typically compact and relatively inexpensive (compared with other traditional imaging modalities) so that devices can be developed to be fully transportable and capable to easily fit either within the surgical room or in its proximity (e.g., in a post-surgical area) without encumbrance.

We present here the first prototype of a compact, fully transportable HSI setup called “HyperProbe1,” which is capable of rapidly selecting at high density, sampling, and spectral resolution (3.5 to 7 nm of bandwidth) any desired wavelength band between 510 and 900 nm and to image a target at a field of view (FOV) of $0.9\times 0.9\;{\mathrm{mm}}^{2}$ with up to 79 spectral bands, in less than 5 min. HyperProbe1 has the capability to image broadband reflected light from fresh biopsies and to reconstruct maps of their optical properties, as well as to quantify the content of biomarkers of interest within the examined tissue (such as the two forms of hemoglobin and the changes in the oxidation state of CCO) via fast spectral unmixing algorithms. We provide a full technical characterization of the performances of HyperProbe1 and a proof of concept of its application on samples of freshly excised glioma from surgical biopsies at different 2021 World Health Organization (WHO) gradings.^14^ The success of HyperProbe1 in providing quantitative biochemical analysis and mapping of surgical biopsies can pave the way to a novel, fast, and heightened methodology to perform *in situ* histopathological screening right after surgery, without the need for any manipulation or degradation of the samples.

## 2 Material and Methods

### 2.1 HyperProbe1 System

**Fig. 1:**
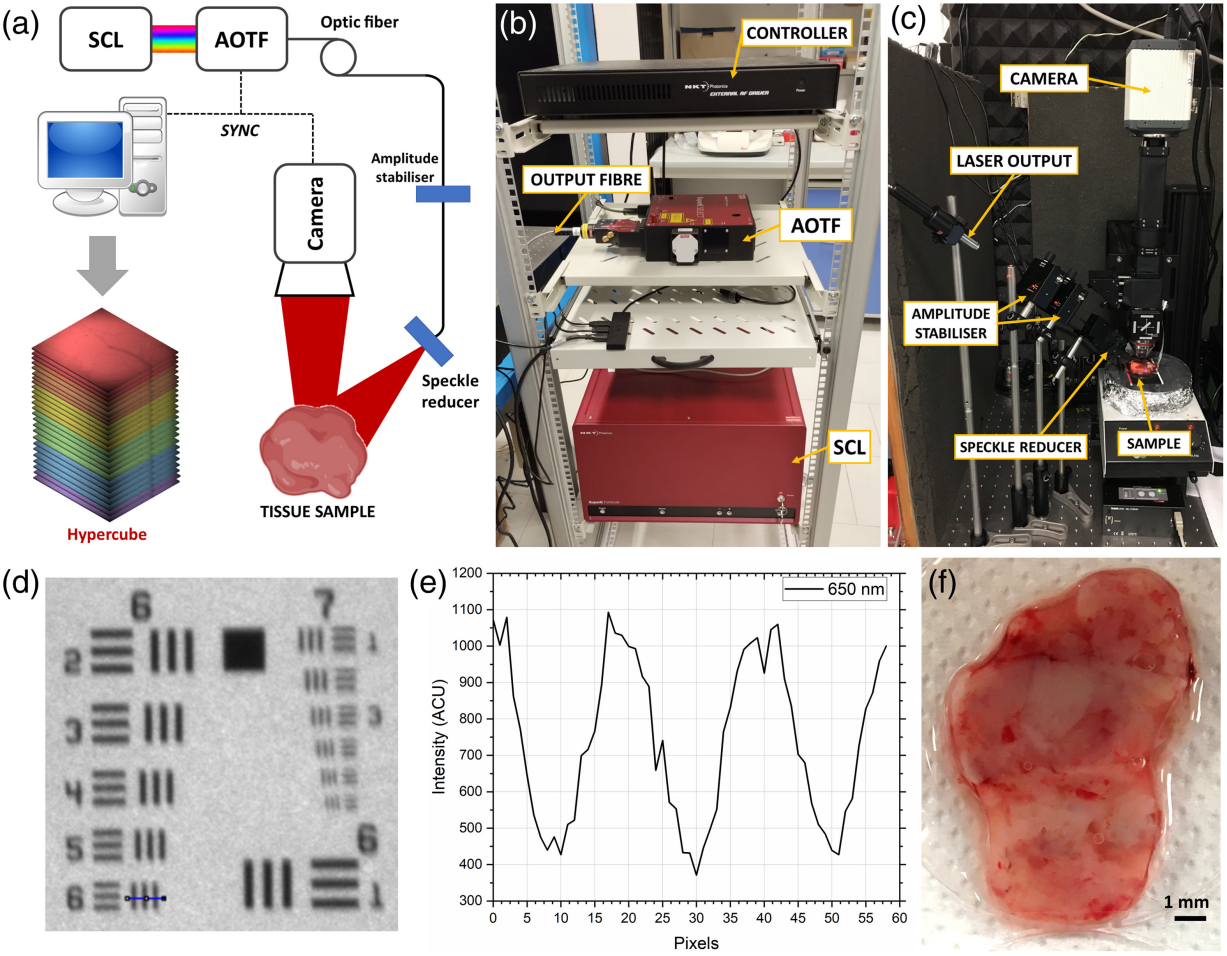
(a) Schematics of the HyperProbe1 with all its components. (b) Picture of the spectral illumination side of HyperProbe1, including the SCL source, the AOTF, and the controlling devices. (c) Picture of the imaging and detection side of HyperProbe1, depicting the illumination output, the amplitude stabilizers, the speckle reducer, and the camera. (d) Result of the imaging tests on the USAF1951 target with HyperProbe1 at 650 nm. (e) The smallest resolved line pair (group 6, element 6) is highlighted in blue and its corresponding line profile that shows minimum FWHM separation (4.38  μm). (f) Example of a sample of excised glioma tissue obtained from the surgical biopsies.

HyperProbe1 is an HSI system based on spectral scanning acquisition mode, where the target is illuminated in rapid sequence at each selected wavelength band, and a full-frame image is acquired synchronously at each illumination step. All the spectral frames are then stacked together to reconstruct the corresponding 3D hypercube of the target.^8^ A schematic of the configuration of HyperProbe is reported in Fig. 1(a), whereas a detailed list of components is presented in Table 1.

**Table 1:** List of components of HyperProbe1 and their specifications.

Component	Manufacturer and model	Key specifics
SCL	NKT Photonics, SuperK FIANIUM FIR20	Broadband illumination (400 to 2400 nm)
		Maximum total power of 6.5 W
AOTF	NKT Photonics, SELECT VIS-nIR	Broadband selection (510 to 900 nm)
		Spectral resolution of 3.5 to 7 nm (FWHM)
Camera	Hamamatsu, ORCA-Flash 3.0	4.2-MP (2048×2048) CMOS sensor
		6.5-μm pixel size;
		QE up to 82% (Visible and NIR)
		Maximum frame rate of 40 fps
Amplitude stabilizers	Thorlabs, NEL02A/M + NEL03A/M	Amplitude stabilization within ±0.05%
Speckle reducer	Optotune, LSR-3005-6D-NIR	Transmission up to 98%;
Optic fibers	NKT Photonics, SuperK CONNECT	Broadband coverage (400 to 2000 nm)
		High-power throughput (up to 500 mW)
		1-mm core diameter
Objective	Thorlabs, LMM15X-P01	15× reflective objective
		NA = 0.3

SCL, supercontinuum laser; AOTF, acousto-optic tunable filters; CMOS, complementary metal-oxide semiconductor; FWHM, full-width at half maximum; QE, quantum efficiency; NIR, near-infrared; FOV, field of view; NA, numerical aperture.

The illumination side of HyperProbe1 [Fig. 1(b)] is composed of a supercontinuum laser^15^ (SCL; NKT Photonics, SuperK FIANIUM FIR20), generating a coherent, broadband illumination (400 to 2400 nm) at a maximum total power of 6.5 W, and a set of acousto-optic tunable filters (AOTF; NKT Photonics, SELECT VIS-nIR) that selectively filters any desired spectral band between 510 and 900 nm, with a minimum bandwidth (full width at half maximum; FWHM) of 3.5 nm in the visible and a maximum bandwidth of 7 nm in the NIR range.

The filtered light from the AOTF is directed to the sample through an optic fiber delivery system (NKT Photonics, SuperK CONNECT) of 1-mm core diameter, coupled with (1) a pair of laser amplitude stabilizer connected in series (Thorlabs, NEL02A/M, and NEL03A/M), to eliminate intensity noise and maintain illumination stability over time within 0.05% of a selected output power across the whole spectral range; (2) a laser speckle reducer (Optotune, LSR-3005-6D-NIR); and (3) an achromatic doublet lens, to make the beam divergent to obtain an illumination spot of 2 to $3\;{\mathrm{cm}}^{2}$ on the target. Image acquisition at each spectral band is obtained on the imaging side of HyperProbe1 [Fig. 1(c)] by the use of a complementary metal-oxide semiconductor (CMOS) camera (Hamamatsu, ORCA-Flash 3.0). The CMOS camera has a sensor size of 2048×2048  pixels, with a pixel size of 6.5  μm, 82% peak quantum efficiency (at 620 nm), and maximum readout rate of 40 fps. It is coupled with a 15× reflective objective (Thorlabs, LMM-15X-P01) and an infinity-corrected, conjugated tube lens (Thorlabs, TTL200-B), to generate an FOV of the target of about $0.9\times 0.9\;{\mathrm{mm}}^{2}$. The whole setup is mounted on a wheeled rack [as depicted in Fig. 1(b)] and can be easily transported within or adjacent to the surgical room, with the illumination probe and imaging side that can be laid on a small breadboard for improved stability.

The HyperProbe1 system was characterized in terms of power emission, spectral, temporal, and imaging performances. The complete characteristics and features of HyperProbe1 are reported in Table 2. HyperProbe1 can scan the entire spectral range of operation (510 to 900 nm) by sampling sequentially up to 79 wavelength bands at 5 nm steps. Each spectral band is also modulated in amplitude by the AOTF, to provide an approximately constant output power on the target of about 200 to 450  μW per band, accounting also for the quantum efficiency (QE) of the camera. This is aimed at maintaining a fixed integration time of the camera for each spectral frame (between 5 and 30 ms, depending on the target), as well as an adequate signal-to-noise ratio (SNR) throughout every acquired spectral frame. The typical acquisition time for an entire hypercube (79 bands) ranges between 1 and 5 min (depending on the selected integration time of the camera), for biological targets. The spatial resolution of HyperProbe1 was assessed for each single spectral band via imaging of a positive resolution test target, the USAF 1951 (Thorlabs, R1L1S1P). For all these imaging assessments, the spatial resolution of HyperProbe1 was found equal to 114  lp/mm, corresponding to the smallest resolvable detail of 4.38  μm [as shown in Figs. 1(d) and 1(e), for 650 nm].

**Table 2:** Technical characteristics and features of HyperProbe1.

Characteristics	Illumination side
Illumination mode	Spectral scanning
Available spectral range	510 to 900 nm
Minimum sampling step size	5 nm
Maximum number of spectral bands	79 (visible and NIR)
Spectral resolution (FWHM)	3.5 nm (visible), 7 nm (NIR)
Average output power per spectral band	∼200 μW (visible), ∼450 μW (NIR)
Power stability over time	±0.05%
**Characteristics**	**Imaging side**
Type of detector	CMOS
Sensor format	2048×2048 pixels
Pixel size	6.5 μm
Spatial resolution	4.38 μm
FOV	$0.9\times 0.9\;{\mathrm{mm}}^{2}$
Frame rate	40 fps (in full format)
Sensitivity (QE)	82% at 620 nm
Typical acquisition time (per spectral frame)	5 to 30 ms
Typical acquisition time (per hypercube)	1 to 5 min

NIR, near-infrared; FWHM, full-width at half maximum; CMOS, complementary metal-oxide semiconductor; FOV, field of view; QE, quantum efficiency.

### 2.2 Sample Preparation and Data Acquisition

HyperProbe1 was used to image a series of fresh surgical biopsies of glioma tissue, to validate its performances in retrieving quantitative information of interest on the composition of samples, as well as to infer pathological characteristics of the tumors akin to what is obtained via traditional histopathological screening. The brain tissue samples involved in the study [an example is shown in Fig. 1(f)] were obtained from fresh surgical excisions of patients taken during routinely performed neurosurgery for brain tumor resection at the Azienda Ospedaliero-Universitaria Careggi (University Hospital of Florence) in Florence. Authorization for the study (Studio ID: 23672 - 23672_BIO) was granted by the Ethical Committee of the Area Vasta Centro Toscana, under Italian law and regulations. Informed consent was collected from each patient involved in the study.

A total number of 11 samples (n=11) were imaged and analyzed with HyperProbe1, to guarantee a degree of statistical robustness in the reconstructed spectra and to investigate specimen variability. The samples were composed of portions of the same tissue removed during the resection that is normally sent to the laboratory for histopathological screening, which classified the type of the tumor based on 2021 WHO gradings.^14^ According to such classification system, gradings of gliomas have been classified as following: Astrocytoma, IDH-mutant 2, 3, 4; Oligodendroglioma, IDH-mutant, and 1p/19q-codeleted 2, 3; glioblastoma, IDH-wildtype 4; diffuse astrocytoma, MYB- or MYBL1-altered 1. This class of information for all the samples is reported in Table 3, providing a broad characterization of various typologies of glioma.

**Table 3:** Classification of the tissue samples investigated with HyperProbe1.

Sample identifier	2021 WHO grading	Additional info
S1	4	
S2	4	
S3	3	Discarded due to fragmented size
S4 (FOV1)	4	Two separate FOVs were acquired on the same sample
S4 (FOV2)	4	Two separate FOVs were acquired on the same sample
S5	4	Labeled with fluorescein
S6	2	Discarded due to the presence of light interference
S7	2	Possibly shifting to a higher grade (3)
S8	3	Labeled with fluorescein, presence of coagulated tissue
S9	4	Labeled with fluorescein, but negative
S10	2	Labeled with fluorescein
S11	4	Labeled with fluorescein, but negative

FOV, field of view.

Two samples were discarded from the analysis: sample S3 was a biopsy composed of very small and dispersed fragments of brain tissue (2 to 3 mm each at most), and due to their dimensions, the acquired spectra appeared very flat (we hypothesize a large influence of partial volume effect); conversely, sample S6 presented ambiguous patterns due to potential light interference at some wavelengths. In addition, for sample S4, due to its slightly larger dimensions than the rest of the biopsies (5 to 6 cm), it was decided to acquire two separate FOVs on the same tissue to further analyze variability within subjects. Finally, samples S8, S9, S10, and S11 were marked with fluorescein during the surgery, albeit S9 and S11 were confirmed to be negative for fluorescent emission.

For the HSI data acquisition, a portion of the glioma samples (average size of 2 to 3 cm) was pre-emptively washed in phosphate-buffered saline (PBS) to eliminate blood and other unwanted residuals, then imaged on its surface with HyperProbe1 (79 wavelength bands at 5-nm steps between 510 and 900 nm) within 1 h after excision. A thin glass coverslip was placed over each sample to flatten its top surface for uniform focusing of the FOV, and a dark absorbing material was placed at the bottom to avoid any potential reflection of the light back into the tissue. The acquisition of a single hypercube for every sample was typically less than 5 min, a short enough duration to ensure that the tissue had not deteriorated nor oxidized during the imaging.

### 2.3 Data Processing and Spectral Unmixing Analysis

Reflectance hypercubes R(x,y,λ) for each biopsy sample were reconstructed by normalizing the hyperspectral data of the reflected light intensity I(x,y,λ) acquired with HyperProbe1 with reference hypercubes W(x,y,λ) obtained using a white calibration standard (Labsphere, Spectralon^®^ 5″), after subtraction of dark counts D(x,y,λ), taken with no spectral illumination and the objective covered, and by weighting the latter two datasets for the ratios of their corresponding integration times t of the camera used during the acquisition. Every dataset was acquired while any ambient light was switched off. The formula for the reconstruction of the reflectance hypercubes is

$$R(x,y,\lambda )=\frac{I(x,y,\lambda )-\frac{t_{I}}{t_{D}}D(x,y,\lambda )}{\frac{t_{I}}{t_{W}}W(x,y,\lambda )-\frac{t_{I}}{t_{D}}D(x,y,\lambda )}$$ (1)

where $t_{I}$, $t_{D}$, and $t_{W}$ are the camera integration times for each frame of the intensity, dark, and white hypercubes, respectively.

A fast, spectral unmixing approach based on modified Beer-Lambert’s law (MBLL) was used to infer the differences in the molecular composition of the biopsies, as described in Ezhov et al.^16^ These differences were quantified with respect to sample S1, using the average spectral reflectance of the central area of the sample as baseline spectrum. Computational time to analyze a full dataset from each biopsy was about 2 to 3 min with two AMD EPYC 7452 32-Core processors.

**Fig. 2:**
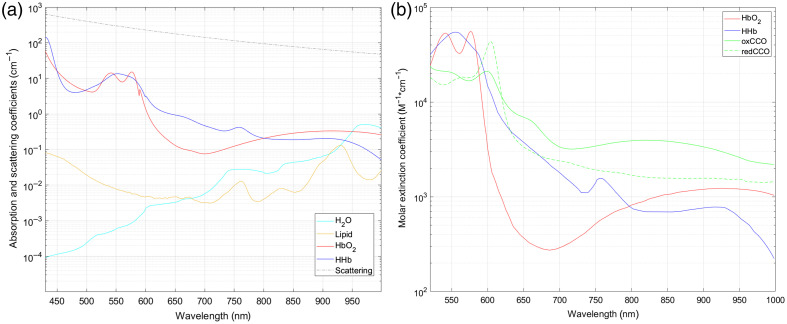
(a) Absorption coefficients for ${\mathrm{HbO}}_{2}$, HHb, lipids and water, and scattering coefficient of generic brain tissue in the visible and NIR range (150  g/L concentration of Hb in the blood and blood volume content in generic brain tissue equal to 5% are assumed).^8^^,^^19^ (b) Molar extinction coefficients of ${\mathrm{HbO}}_{2}$, HHb, oxCCO, and redCCO in the visible and NIR range.^8^^,^^17^

We then compared the inferred compositions for two different scenarios: (1) by fitting the whole measured wavelength range (from 510 to 900 nm) and (2) by fitting only the NIR portion of the available spectrum (in our case, from 740 to 900 nm). For the latter, we expected the major absorbing chromophores to be oxygenated (${\mathrm{HbO}}_{2}$) and deoxygenated (HHb) hemoglobin, the oxidized (oxCCO) and reduced (redCCO) forms of cytochrome-c-oxidase (CCO), as well as water and lipids.^8^^,^^17^^,^^18^ The spectral signatures of the chromophores targeted by the analysis are depicted in Figs. 2(a) and 2(b). In the visible range, we also assumed the presence of additional chromophores, specifically the oxidized and reduced forms of cytochrome-b (Cyt-B) and cytochrome-c (Cyt-C), due to their involvement in the metabolic processes.^17^

The inferred compositions, in the forms of either concentrations or volumetric contents, are in units (mM/cm) and (${\mathrm{cm}}^{-1}$), respectively, as we used unitary pathlength (1 cm) in our experiments. We have previously seen that a quasi-constant pathlength only effectively scales the concentrations, at least in the NIR range,^16^ and is therefore sufficient for the preliminary task of attempting to distinguish biopsies of different tumor gradings.

### 2.4 Monte Carlo Simulations of Penetration Depth in Tissue

A 3D, *in silico*, optical and geometrical model of brain biopsy was designed to assess and quantify the depth of penetration of light in the tissue, at the various spectral bands of HyperProbe1.^9^ This was done in MATLAB using a voxel-based Monte Carlo (MC) simulation software. The software chosen was Monte Carlo eXtreme (MCXLAB),^20^^,^^21^ which simulates photon transport within a 3D, voxel-based model with arbitrary optical properties. The simulations were carried out on a desktop computer with an Intel Xeon W5-3425 and two RTX 4090 GPUs.

The simulated geometry consisted of a homogeneous, semi-infinite slab of gray matter with a thickness of 0.5 cm (taking into account the maximum thickness recorded among the investigated samples). Isotropic voxels were used, with dimensions of 0.05 mm. This was chosen as it offered an acceptable trade-off between accuracy and computational performances. A black absorbing layer of 0.005 cm thickness with a significantly higher absorption coefficient (in the order of $10^{6}\;{\mathrm{cm}}^{-1}$) was placed at the bottom of the slab, to represent the absorbing material used below the biopsies. Fresnel reflection was implemented at the top and bottom boundaries of the model.^20^

The reduced scattering coefficient $\mu_{s}^{\prime }$ of the gray matter of the model was adopted from Jacques et al.^18^ and was varied with wavelength according to the following equation:

$$\mu_{s}^{\prime }=a{\left(\frac{500\;\mathrm{nm}}{\lambda }\right)}^{-b},$$ (2)

where $a=40.8\;{\mathrm{cm}}^{-1}$ and b=3.089. From $\mu_{s}^{\prime }$, the scattering coefficient $\mu_{s}$ was calculated as an input for MCXLAB with the equation:

$$\mu_{s}=\frac{\mu_{s}^{\prime }}{1-g},$$ (3)

where g is the anisotropy factor of grey matter standing at 0.85 and chosen to be constant, as its variation with wavelength has been demonstrated to be minimal for cerebral tissue.^22^ Furthermore, the refractive index of the biopsy model was set to 1.36.^18^

Finally, the 3D model of brain biopsy was assumed to be composed of the most absorbing and scattering tissue chromophores, i.e., water, lipids, ${\mathrm{HbO}}_{2}$, HHb, oxCCO, and redCCO.^9^ Thus, the total absorption coefficient $\mu_{a}$ of the model was determined using the equation:^9^^,^^18^

$$\mu_{a}=W\cdot \mu_{a,H_{2}O}+F\cdot \mu_{a,\text{fat}}+\ln\;10\cdot C_{\mathrm{HHb}}\cdot \varepsilon_{\mathrm{HHb}}+\ln\;10\cdot C_{{\mathrm{HbO}}_{2}}\cdot \varepsilon_{{\mathrm{HbO}}_{2}}+\;\ln\;10\cdot C_{\mathrm{oxCCO}}\cdot \varepsilon_{\mathrm{oxCCO}}+\ln\;10\cdot C_{\text{redCCO}}\cdot \varepsilon_{\text{redCCO}},$$ (4)

where W and F are the water and lipids volumetric contents, respectively; $\mu_{a,H_{2}O}$ and $\mu_{a,\text{fat}}$ are the absorption coefficients of water and lipid, respectively; $C_{\mathrm{HHb}}$, $C_{{\mathrm{HbO}}_{2}}$, $C_{\mathrm{oxCCO}}$, and $C_{\text{redCCO}}$ are the molar concentrations of HHb, ${\mathrm{HbO}}_{2}$, oxCCO, and redCCO, respectively; and $\varepsilon_{\mathrm{HHb}}$, $\varepsilon_{{\mathrm{HbO}}_{2}}$, $\varepsilon_{\mathrm{oxCCO}}$, and $\varepsilon_{\text{redCCO}}$ are the molar extinction coefficients of HHb, ${\mathrm{HbO}}_{2}$, oxCCO, and redCCO, respectively. W, F, $C_{\mathrm{HHb}}$, $C_{{\mathrm{HbO}}_{2}}$, $C_{\mathrm{oxCCO}}$, and $C_{\text{redCCO}}$ are derived from Giannoni et al.,^9^ whereas the absorption coefficients $\mu_{a,H_{2}O}$ and $\mu_{a,\text{fat}}$ [graphed in Fig. 2(a)], as well as the molar extinction coefficients $\varepsilon_{\mathrm{HHb}}$, $\varepsilon_{{\mathrm{HbO}}_{2}}$, $\varepsilon_{\mathrm{oxCCO}}$, and $\varepsilon_{\text{redCCO}}$ [graphed in Fig. 2(b)] were derived from Giannoni et al.^9^ and Prahl et al.^19^ Table 4 summarizes the main composition of the 3D *in silico* model of brain biopsy used for the MC simulations, based on human gray matter.

**Table 4:** Composition of the 3D *in silico* model of brain biopsy used for the MC simulations.

Model composition	Values
Water content, W	70%
Lipid content, F	10%
Molar concentration of HHb, $C_{\mathrm{HHb}}$	56.7 μM
Molar concentration of ${\mathrm{HbO}}_{2}$, $C_{{\mathrm{HbO}}_{2}}$	56.7 μM
Molar concentration of oxCCO, $C_{\mathrm{oxCCO}}$	1 μM
Molar concentration of redCCO, $C_{\text{redCCO}}$	4 μM

HHb, deoxyhaemoglobin; ${\mathrm{HbO}}_{2}$, oxyhemoglobin; oxCCO, oxidized cytochrome-c-oxidase; redCCO, reduced cytochrome-c-oxidase.

A planar, divergent light source was positioned above the 3D biopsy model to simulate the illumination of the gray matter slab at the same wavelengths of operation of the HyperProbe1 (500 to 900 nm, at 5-nm sampling). The direction of the illumination was normal the z axis. A total of $10^{7}\text{ photons}$ were simulated for each wavelength band, with emission power equal to 1 mW.

## 3 Results

### 3.1 Results of the Monte Carlo Simulations of Penetration Depth in Tissue

**Fig. 3:**
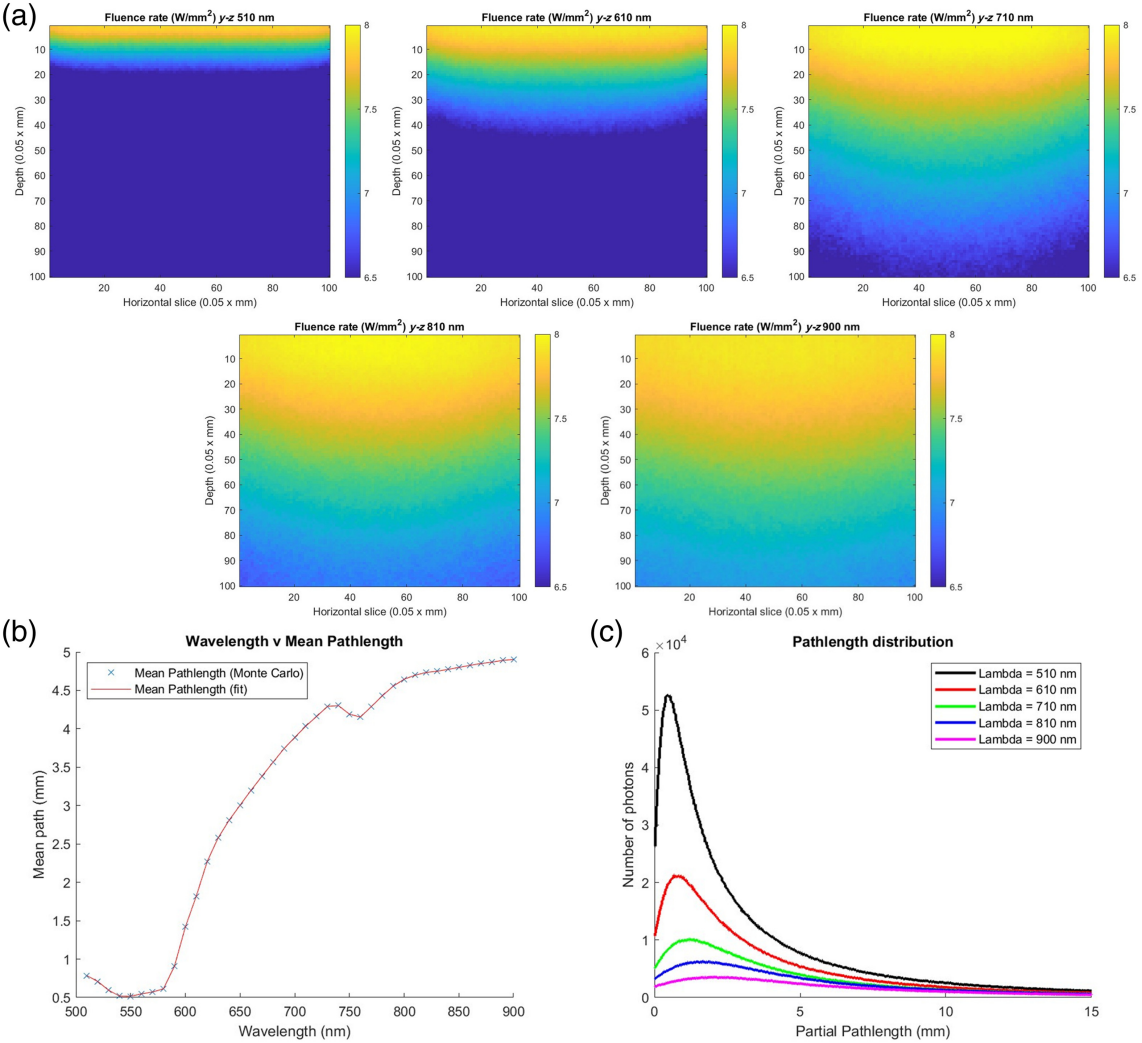
(a) Simulated fluence rates within the 3D cerebral biopsy model at different wavelengths (the plots are in a logarithmic scale and the absorbing layer was excluded from the plots to maintain a visible contrast). The distribution of the fluence showed a noticeably higher penetration of the light at the NIR wavelengths. (b) Simulated mean photon pathlength within the 3D biopsy model, as a function of wavelength. (c) Partial pathlength distributions of the photons simulated within the 3D biopsy model at various wavelengths (as previously, the absorbing layer was not included in this figure due to a negligible number of photons passing through it).

Simulated fluence rates for each wavelength were obtained from the MC simulations, allowing one to visualize and assess the spatial distribution of the photons at the different spectral bands within the 3D model. Examples of these distributions are depicted in Fig. 3(a), demonstrating increasing depth of penetration of the light within the tissue for longer and longer wavelengths. The depth of penetration of the photons in the model of biopsy ranged from 10 to 15% of its thickness (about 0.5 to 0.75 mm) for the visible light, up to almost the whole size of the sample (5 mm), for the NIR light.

To further investigate the relationship between wavelength and penetration depth of the light, the mean pathlength across the tissue of the simulated photons was estimated from the MC simulations for each spectral band, as reported in Fig. 3(b). The results further demonstrate that the mean pathlength traveled by the photon within the tissue increases as the wavelength of the light gets longer, up to about 5 mm (at 900 nm). Nonetheless, this gradual increase is not homogeneously continuous, as local extrema are identifiable in Fig. 3(b), corresponding to the peaks of absorption of hemoglobin at about 530 to 570 nm and 770 nm [as visible in Fig. 2(a)].

Finally, distributions of the partial pathlengths of the simulated photons were estimated for each wavelength of HyperProbe1: Fig. 3(c) depicts examples of these distributions across the full range of the system (the absorbing layer at the bottom is excluded). The results, together with the outcomes previously illustrated, demonstrate empirically that HyperProbe1 does not simply map the optical properties of the investigated tissue on its visible surface, but actually reconstructs an integrated distribution on 2D of the spectral features of the biopsy samples across their entire 3D volume, thanks to the use of both visible and NIR light with different penetration capabilities.

### 3.2 Qualitative and Comparative Evaluation of the HyperProbe1 Data

**Fig. 4:**
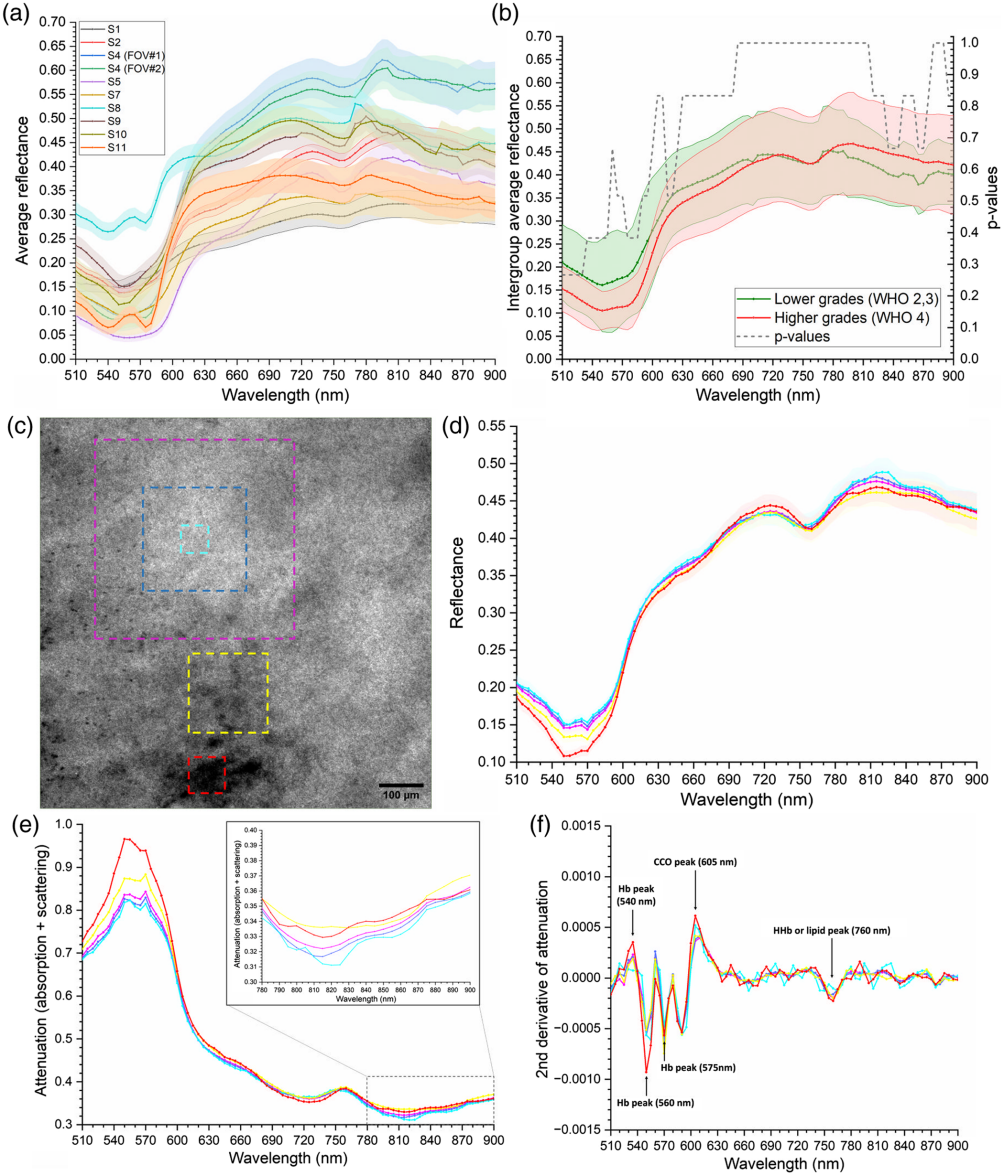
(a) Intercomparison of averaged reflectance spectra over the entire imaged FOVs of each biopsy sample. (b) Comparison between average reflectance spectra grouping LGG (WHO 2, 3) against HGG samples (WHO 4), with resulting p-values from statistical analysis on differences between each wavelength. (c) Processed spectral image from HyperProbe1, at 560 nm, of HGG (WHO grade 4) biopsy sample S2, with highlighted, selected ROIs in the FOV in which average reflectance spectra were calculated. (d) Example of average reflectance spectra in the corresponding ROIs of the biopsy sample S2. (e) Example of average attenuation spectra in different ROIs of biopsy sample S2, with the portion within the NIR range 780 to 900 nm enlarged. (f) Example of the second derivative of the average attenuation spectra in different ROIs of biopsy sample S2.

Preliminary qualitative evaluation of the data collected with HyperProbe1 on the cerebral *ex vivo* tissue was conducted, to assess both heterogeneity in the spectra within the same sample, as well as spectral variability between all the biopsies. For intercomparison across the various samples described in Table 3, each processed reflectance hypercube $R(x,y,\lambda {)}_{n}$, for n=S1…S11, was averaged spatially over its entire FOV, to obtain a single averaged reflectance spectrum for each biopsy. All these reflectance spectra are shown altogether in Fig. 4(a). Overall, the average reflectance spectra between samples share similar trends, reporting low reflectance in the visible range [where absorption from hemoglobin is at its highest, as per Fig. 2(a)], which then tends to increase gradually toward the NIR range beyond 600 nm, where scattering becomes predominant. However, significant variability in both magnitudes of the spectra in the same regions and in their local features are also present, differentiating the signatures of the various samples. Such an aspect is further highlighted by averaging the abovementioned spectra according to their 2021 WHO grading: lower grade glioma (LGG) samples (WHO grades 2 and 3) were grouped together and the mean of their average reflectance spectra across the whole FOVs was compared against the same mean for all the higher grade glioma (HGG) samples (WHO grade 4), as reported in Fig. 4(b). The decision for this grouping is according to the most recent (2021) WHO grading^14^ and current state of the art in histopathological practice,^23^ where gliomas are grouped molecularly based on their isocitrate dehydrogenase (IDH) and 1p/19q status so that grade 4 glioblastomas (IDH-wildtype) are defined as higher grade and clearly separated from the biologically and clinically more benign IDH-mutant, and 1p/19q-codeleted, lower grade gliomas (defined as WHO grades 2 and 3). Differences in the means between LGG and HGG samples are indeed visible, regarding both local spectral trends as well as magnitude. However, statistical analysis via Mann-Whitney U-test was performed on the grouped means, obtaining p-values for each measured wavelength all equal to or higher than 0.26 [as reported in Fig. 4(b)], which indicates that there is no single-independent wavelength that can distinguish tumor grading (thus supporting the hypothesis of performing spectral unmixing).

A number of different regions of interest (ROI) were selected on the reflectance hypercubes R(x,y,λ) to calculate average reflectance spectra, including different sizes and portions of the FOV reporting identifiable visual features, such as blood clusters. This was done to investigate potential spatial changes in the reflectance spectra across the FOV of the samples. Figure 4(c) shows an example of a single, processed reflectance spectral image (as described in Sec. 2.2) collected with HyperProbe1 for arbitrary HGG (WHO grade 4) sample S2, at the bandwidth centered at 560 nm, whereas Fig. 4(d) depicts the corresponding average reflectance spectra. The comparison of the reflectance spectra for each sample on different ROIs highlights significant (from 0.0017 to 0.0378 average root mean square deviation (RSMD) for the reflectance curves across all samples) differences in their shapes and trends, as visible for the model case of S2 in Fig. 4(d), for two specific spectral ranges: (1) between 510 and 660 nm and (2) between 780 and 880 nm. By contrast, outside of these ranges, the remainder of the spectral signatures displays a homogeneous distribution of the optical properties of the *ex vivo* samples across the entire FOVs. The largest differences (0.0378 of average RSMD across samples) are reported for the ROIs where accumulation of the blood is clearly visible. For the first mentioned range (510 to 600 nm), such results could be correlated to the presence and strong influence of the visible peaks of hemoglobin absorption [Fig. 2(a)]. The influence of the absorption of hemoglobin could also be connected to the reported differences in the spectra for the second-mentioned range (780 to 880 nm), which is characterized by a broad peak of absorption from ${\mathrm{HbO}}_{2}$. However, differences are identified in the biopsy samples also for ROIs not including visible blood clusters: this could then be connected to local differences in the concentrations of CCO, as the identified range overlaps with the NIR absorption peak of the latter [Fig. 2(b)], as well as for the increasing weight of the absorption of water and lipids toward the end of the NIR range [Fig. 2(a)].

For a more direct comparison between the spectral signatures of the biopsies reconstructed with HyperProbe1 and the pure optical signatures of the chromophores of interest reported in Figs. 2(a) and 2(b), we calculated the attenuation spectra A(x,y,λ), associated with the contribution of optical absorption and scattering in the tissues, from the reflectance spectra R(x,y,λ) in the same ROIs of the samples, using the formula: $A(x,y,\lambda )=-\log_{10}(R(x,y,\lambda ))$. Figure 4(e) reports, for instance, the average attenuation spectra of the same HGG sample S2 and the previously selected ROIs. In the range 510 to 600 nm, the attenuation spectra of the brain tissue samples correlate with the combined profiles of the absorption spectra of ${\mathrm{HbO}}_{2}$ and HHb, with noticeable peaks at around 545 to 555 nm and at 575 nm. Another peak is also identified in all samples at around 755 to 760 nm, overlapping the equivalent one from the absorption spectra of HHb. In all samples, attenuation in the range 510 to 600 nm is reported to increase gradually when shifting from ROIs with no visible accumulations of blood toward ROIs that include the latter at different degrees of covering [as visible for the case of S2 in Fig. 4(d)]. Figure 4(e) highlights and enhances the visualization of the attenuation spectra from S2 in the range between 780 and 900 nm, where differences are reported for all the ROIs regardless of the presence of any discernible spatial feature or difference in contrast. This is occurring largely across all the analyzed samples of surgical biopsies. In particular, localized peaks of attenuation corresponding to about 840 nm are identified in a number of ROIs in the samples, where the optical absorption of oxCCO is also at its highest. The reported discrepancies among concentric ROIs of different sizes in relatively homogenous areas of the *ex vivo* tissue could be linked to either local variation in the abovementioned chromophore, as suggested by the overlapping of the peaks, or to partial volume effects connected to changes in the optical pathlength traveled by the lights within the sampled regions.^9^

Finally, to further highlight the major features in the average attenuation spectra, a spectral derivative analysis was performed, by taking the derivative of the average attenuation spectra from the biopsies twice with respect to the wavelength (second order derivative), via a finite divided difference approximation algorithm. Such a procedure has the advantage of enhancing sharp contribution to absorption (such as predominant peaks that may be overlapping with each other’s), of helping identify maximum attenuation wavelengths for broad peaks, as well as dampening the effect of scattering in the data.^24^ As an exemplary case, Fig. 4(f) reports the second-order derivative of the average attenuation spectra for the same HGG sample S2 and the previously selected ROIs. Second-order features connected to the visible absorption peaks of both forms of hemoglobin emerge even more significantly from such analysis [located at about 540, 560, and 575 nm, as reported in Fig 2(a)], as well as to the NIR peak at 760 nm, which can be correlated to either HHb or lipid absorption [as seen again in Fig. 2(a)]. In addition, a large second-derivative peak can also be identified at 605 nm, which may be related to the overlapping visible peaks of both oxCCO and redCCO^17^ [as seen in Fig. 2(b)].

### 3.3 Quantitative Biochemical Analysis of the HyperProbe1 Data with Spectral Unmixing

**Fig. 5:**
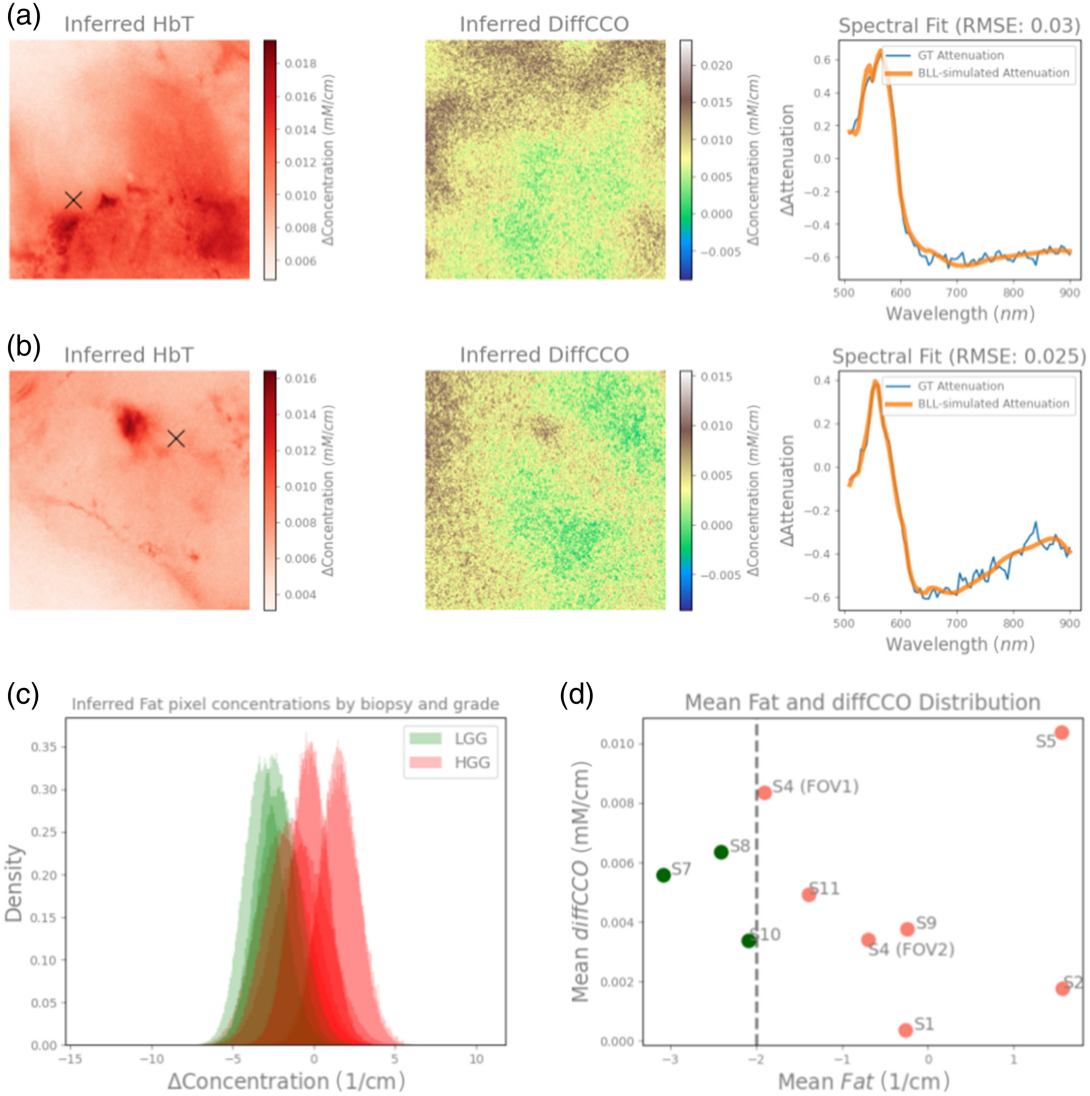
Inferred HbT and diffCCO concentration maps of HGG S4 FOV#1 (a) and LGG grade S10 (b) samples fitting the whole measured wavelength spectrum (510 to 900 nm), and a model fitting of the observed attenuation for the marked pixel in the HbT image is shown in the third column for both biopsies. (c) Histogram showing probability density distribution of inferred lipid volumetric content of each pixel across different LGG (displayed in green) and HGG (displayed in red) grade samples. (d) Distribution means of the lipid content (reported on x-axis) and diffCCO concentrations (reported on y-axis) suggest that lipid mean content could be able to distinguish the grading of all samples, whereas no apparent separation is visible for the inferred mean diffCCO concentrations.

As mentioned in Sec. 2.2, we compared the inferred compositions of the expected chromophores from the spectral unmixing algorithm in all the biopsy samples, for two different fitting scenarios: in the whole range from 510 to 900 nm and in the NIR portion of the spectrum from 740 to 900 nm. For the first scenario, we obtained satisfactory spectral fits matching the measured attenuation: an example is depicted in Figs. 5(a) and 5(b), for HGG sample S4 in FOV#1 (WHO grade 4) and LGG sample S10 (WHO grade 2), showing also the reconstructed quantitative maps for the total concentration of hemoglobin (HbT), given as the sum of ${\mathrm{HbO}}_{2}$ and HHb, as well as for the concentration of differential CCO (diffCCO), given as the difference between the concentrations of oxCCO and redCCO.^8^^,^^17^ Blood clusters are resolved with high resolution, due to the hemoglobin peaks in the 500 to 600 nm range and the expected high concentration and absorbance of hemoglobin (compared with the other known chromophores). Similar well-matching spectral fits via MBLL are also found across all patients, as we report the root mean square error (RMSE) means across all pixels and across all patients to be in the range 0.017 to 0.039, which is of similar magnitude to the exemplary RMSEs reported in Fig. 5.

A preliminary attempt to classify the biopsy samples from the inferred hyperspectral results was also performed. As shown in Figs. 5(c) and 5(d), we found that predicted lipids content allows us to separate LGG (grades 2 and 3) biopsies from HGG biopsies (grade 4). Even though we observed significant overlap between the different distributions (with an overlap coefficient of 49.96% assuming normal distributions), we generally noticed a trend of higher differential lipids content in HGG biopsies, with a mean of $-0.466\;{\mathrm{cm}}^{-1}$. Conversely, the LGG samples were found to have a mean lipid content of $-2.53\;{\mathrm{cm}}^{-1}$. The distinction between the two gradings for all samples was possible by computing the means across all the biopsies and using the $-2\;{\mathrm{cm}}^{-1}$ lipid content difference threshold, as shown in Fig. 5(d). This difference in means of the tumor grades was also found to be statistically significant via the Mann-Whitney U-test (p=0.017), testing for equal means. Conversely, we did not find evidence of CCO or hemoglobin (established biomarkers for cellular metabolism and hemodynamics, respectively^8^) to be able to separate gradings of glioma samples in the whole range 510 to 900 nm [as it can be seen in Fig. 5(d), for CCO]. The overlap coefficient between LGG and HGG samples was found to be considerably larger, with 81.2% and 84.6% for inferred diffCCO and ${\mathrm{HbO}}_{2}$ concentrations, and statistical differences in grading were not observed (p=0.84 and p=0.99, respectively). On a final note, as seen in Fig. 5(d), by considering both variables (lipid content and diffCCO concentration) on the 2D distribution plot, an oblique line could arguably even better separate the two glioma grades. A larger sample size will be required to test such more complex hypotheses further.

**Fig. 6:**
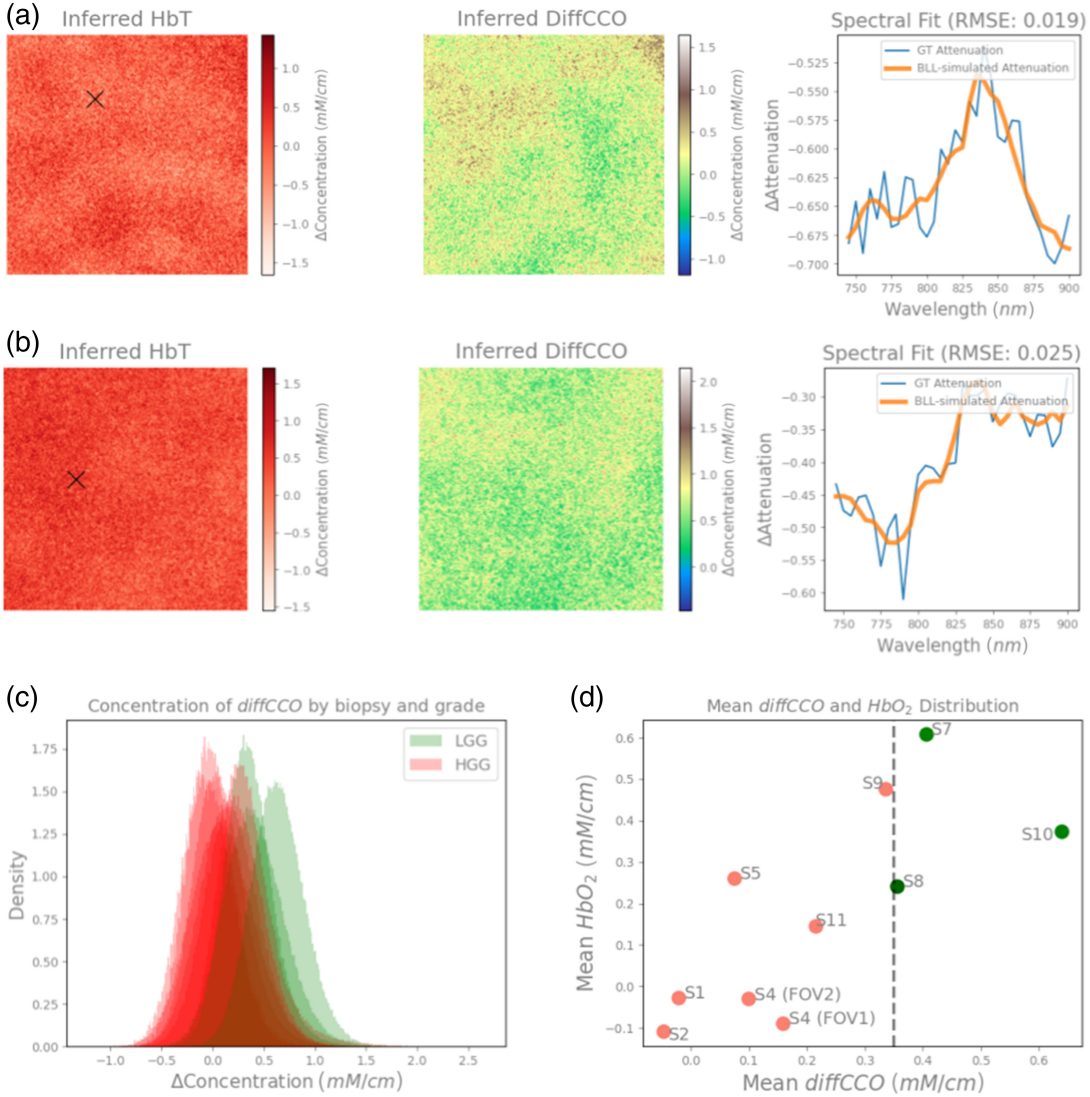
Inferred HbT and diffCCO concentration maps of HGG S4 FOV#1 (a) and LGG S10 (b) samples fitting exclusively the NIR range (740 to 900 nm), and a model fitting of the observed attenuation for the marked pixel in the HbT image is shown in the third column for both biopsies. (c) Histogram showing probability density distribution of inferred diffCCO concentrations of each pixel across different LGG (displayed in green) and HGG (displayed in red) grade samples. (d) Distribution means of diffCCO and ${\mathrm{HbO}}_{2}$ concentrations suggest that these parameters could be able together to distinguish lower and higher grade samples of glioma tissue, with diffCCO being the most accurate, across all the investigated samples.

In the second scenario, we estimated inferred compositions within the biopsy samples using exclusively the NIR range between 740 and 900 nm, which was chosen to target chromophores that are known for their characteristic absorption profiles in such range, particularly oxCCO [as seen in Fig. 2(b)]. Indeed, MBLL has been commonly employed in this specific NIR range to infer differences in metabolic activity using CCO as a biomarker.^8^^–^^10^^,^^17^ As shown in Figs. 6(a) and 6(b), we again fit the observed signal qualitatively well: the inter-biopsy mean RMSE errors are found to be in the range 0.0151 to 0.296, i.e., the spectral fits of all biopsies can be expected to be similar as observed in Figs. 6(a) and 6(b), as for the previous scenario, albeit slightly worse due to the expected reduction in SNR at the latter end of the measured spectrum, where scattering of light becomes predominant. Notable loss in the resolution of various blood clusters can be observed, which was also expected due to the exclusion of the 510 to 600 nm range with larger hemoglobin absorption peaks.

**Fig. 7:**
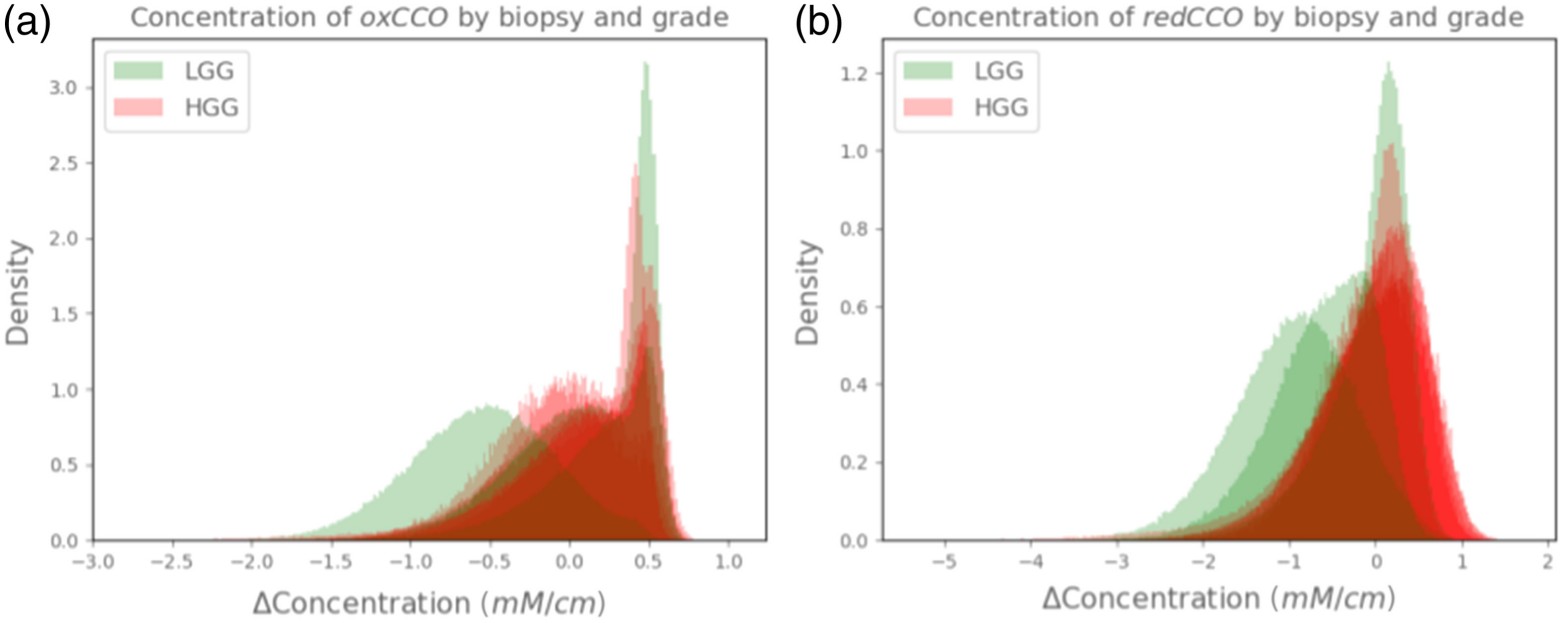
Histogram showing probability density distribution of inferred oxCCO (a) and redCCO (b) concentrations of each pixel across different LGG (displayed in green) and HGG (displayed in red) grade samples. Neither the inferred oxCCO nor redCCO density distributions using the NIR range (740 to 900 nm) suggest being able to differentiate tumor grading, contrary to the diffCCO mean concentrations.

Interestingly, we observed significant differences in the inferred molecular concentrations across biopsies of LGG and HHG that do not match the ones highlighted by the spectral analysis on the whole wavelength range: e.g., in this scenario, the inferred lipid contents were not able to distinguish between the different grades of the samples. We reported an overlap coefficient of 77.1% and no statistical differences in the concentration means for lipids in this scenario (p=0.84). However, we observed average concentration differences of metabolic diffCCO between LGG and HGG samples, with HGG samples showing lower diffCCO mean concentrations, as seen in Figs. 6(c) and 6(d). The diffCCO concentration threshold at 0.35  mM/cm was able to distinguish all LGG and HGG samples by computing the intra-biopsy diffCCO concentration mean (p=0.017), despite visible overlap (with overlap coefficient of 61%) between the distributions as seen in Fig. 6(c). Singularly, we also reported that both oxCCO and redCCO individually are seemingly not able to distinguish biopsy grading, as we find overlap coefficients of 84.6% and 73.3%, and no statistically significant differences (p=0.99 and p=0.12, respectively). This is shown in Fig. 7 plotting probability density distributions of each pixel across all biopsies, divided between LGG and HGG. Only the difference in diffCCO between the two inferred concentrations resulted in the suggested distinction of the two classes of tumoral grades.

Furthermore, as depicted in Fig. 6(d), we also found an approximately linear correlation in the 2D domain between the mean differences in concentration of diffCCO and those of ${\mathrm{HbO}}_{2}$: together with a reduced diffCCO mean concentration, HGG samples also present a correlated reduction in the mean concentration of ${\mathrm{HbO}}_{2}$, with the combination of the two biomarkers enhancing the possibility of separating samples grading more effectively.

On a final note, we observed that the HGG sample S9 additionally displays diffCCO characteristics close to LGG samples [as reported in Fig. 6(d)]: we hypothesize that there could be different (possibly concurrent) reasons for this low distinguishability: even though it has not been shown that HGG samples can show characteristics of LGG samples, the opposite (i.e., high grade-typical histological characteristics in low-grade lesions) has recently been reported by Motomura et al.^25^ Thus, in general, we suggest that it might be possible that lower grade lesions could display characteristics of higher grade lesions, although further investigation on this would be needed.

## 4 Conclusions

We presented a novel, transportable HSI device, named HyperProbe1, based on fast spectral scanning via a combination of SCL illumination and AOTF filtering. At full operational performances, HyperProbe1 can scan sequentially up to 79 wavelengths between 510 and 900 nm, with 5-nm sampling and high spectral resolution (3.5 to 7 nm of bandwidth), as well as acquire corresponding spectral images on a $0.9\times 0.9\;{\mathrm{cm}}^{2}$ FOV at high spatial resolution (4.38  μm), in less than 5 min.

Furthermore, we also provided a preliminary assessment of the data acquired and analyzed with HyperProbe1 on a number (n=11) of fresh surgical samples of glioma from patients at different WHO gradings, including both lower grade (WHO grades 2 and 3) and higher grades (WHO grade 4) tumors. The initial findings demonstrated the capability of the device to reconstruct quantitative maps of the distribution of the concentrations of various chromophores of interest, in particular hemoglobin and CCO (both established biomarkers for tissue hemodynamics and metabolism, respectively), as well as lipids volumetric content. We also looked at differences in the inferred contributions of these biomolecules between LGG and HGG biopsies that may suggest a way to distinguish between the two and thus potentially provide the basis for an HSI-based methodology for tumor classification. We found indeed significant differences between mean lipid contents across samples that could potentially be used to distinguish tumor grading when fitting over the entire work range (510 to 900 nm). In particular, HGGs presented higher mean lipid content than LGGs: a plausible biological interpretation of this could be connected to substantial lipid storage for lipid metabolism in higher-grade gliomas, a known characteristic feature of GBM.^26^^,^^27^ Among various metabolic alterations we can appreciate in gliomas, lipid metabolism reprogramming is one of the most predominant and has a direct impact on the metabolic plasticity of gliomas and glioblastomas. A recent systematic review by Rashid et al.^28^ has demonstrated that significant lipid alteration is pivotal for gliomas development, reporting also that higher lipid concentration signals were detected in high-grade gliomas relative to low-grade gliomas.

Similarly, we also found that the analyzed HGGs presented inferior concentrations of diffCCO compared with LGGs when fitting the data exclusively in the NIR range (740 to 900 nm): this may suggest a potential metabolic path with NIR light to distinguish lower and higher grade samples. This finding may also emphasize the potential role of diffCCO in providing metabolic differences that lie beyond its critical use in NIR spectroscopy (NIRS) applications.^17^ The exact mechanisms by which these concentration differences in diffCCO arise still remain uncertain: as changes in the concentration of diffCCO are directly proportional to variations in the metabolic activity of the tissue, one would expect an influence of enhanced hypermetabolism in tumors at higher grades. However, HGGs (such as GBMs) are characterized by the unique presence of necrotic tissue as the core part of the lesion, with the hypermetabolic region occurring only at the borders.^29^^,^^30^ Such central necrotic areas, composed almost entirely of dead, i.e., non-metabolic cells, could explain the overall reduced mean concentration of diffCCO in HGG compared to LGG, which instead do not present necrosis. Furthermore, the results showed a direct correlation between the reduction of diffCCO in HGG with a corresponding decrease in ${\mathrm{HbO}}_{2}$, which could also be connected to the hypoxic microenvironment that is specific to this type of brain tumor.^31^^,^^32^ From a biological perspective, HGGs, such as GBMs, are known to be triggered and driven in their proliferation by hypoxic microenvironments within the cerebral tissue.^31^^,^^32^ Such a phenomenon could explain the reduced mean concentration of ${\mathrm{HbO}}_{2}$, which is a biomarker for oxygenation of the brain, and its correlation with the diffCCO because a direct association between oxygen delivery and its metabolic consumption has been strongly established.^33^

Future investigations on a larger and enriched cohort of biopsy samples of various types will be needed to further confirm all these results on a more robust statistical basis. In particular, comparisons with control samples composed of healthy cerebral tissue will also be strongly required for increasing the accuracy and reliability of any prediction.

The goal of HyperProbe1 is to provide a first proof-of-concept application to rapid and quantitative digital histology of *ex vivo* tissue from excised surgical biopsies, in particular of cerebral glioma, by reconstructing quantitative maps of the distributions of chromophores of interest in the tissue via fast spectral unmixing algorithms. In this perspective, we demonstrated that HyperProbe1 can collect and analyze full-range HSI data of light reflectance from surgical biopsies in less than 1 h after their excision (including preparation of the sample and setting up of the measurements), a significantly much faster time than traditional H&E histopathological screening, which normally takes several days up to few weeks. Such a result qualifies HyperProbe1 for an envisioned, future utilization to provide *in situ*, rapid, streamlined, and non-destructive screening of fresh tissue samples, ideally within or just next to the operating theatre. This application could considerably benefit the outcome of the surgical treatment and provide an all-optical advancement to current post-surgical histopathological practice.

From a technological perspective, we also aim at improving the current setup of HyperProbe1 to further enhance its performances: we are currently working on extending the operational spectral range of the device at both ends, by (1) covering the rest of the available visible range below 500 nm and including also part of the near ultraviolet (UV) as well as by (2) expanding the NIR coverage beyond 900 nm. This approach could further enrich the collected spectral data and allow us to potentially target even additional chromophores of interest.^8^

The versatility of HyperProbe1 could also pave the way for potential applications of the instrumentation to virtually any type of surgical and non-surgical biopsy (or other *ex vivo* tissues), as well as even move beyond digital histopathology. In this perspective, HyperProbe1 can also be envisioned as an investigative, high-performance HSI device for preclinical *in vivo* applications: it could be used to explore and tailor features of HSI (such as type and number of wavelengths) that could be translated into clinical settings by specifically engineering more compact, cost-effective, and user-friendly medical devices. Within the framework of the HyperProbe project and consortium,^34^^,^^35^ we indeed aim at translating this technology for its use as a new neuronavigation tool during brain surgery, such as in glioma resection. Such a device would aim at providing an innovative approach to guided neurosurgery, by transforming current practice toward an all-optical, real-time, quantitative, and accurate imaging approach, which could significantly help neurosurgeons, enhance the efficacy of the treatment, and ultimately improve the life expectancy of the patients.

## Data Availability

In support of open science, the data presented in this article are publicly available on Zenodo at https://zenodo.org/records/10908359. Similarly, all our spectral unmixing code is available on GitHub at https://github.com/HyperProbe/SpectraFit.
